# Effects of Positive End-Expiratory Pressure on Intraocular Pressure during One-Lung Ventilation in the Lateral Decubitus Position—A Prospective Randomized Trial

**DOI:** 10.3390/medicina58070940

**Published:** 2022-07-15

**Authors:** Yong Shin Kim, Kwon Hui Seo, Yeon Soo Jeon, Jang Hyeok In, Hong Soo Jung, Yoo Jung Park, Eun Hwa Jun, Eunju Yu

**Affiliations:** 1 Department of Anesthesiology and Pain Medicine, St. Vincent’s Hospital, College of Medicine, The Catholic University of Korea, Suwon 16247, Korea; aneskim@catholic.ac.kr (Y.S.K.); likewinds@catholic.ac.kr (Y.S.J.); ijangh@hanmail.net (J.H.I.); flood1@naver.com (H.S.J.); pyj@catholic.ac.kr (Y.J.P.); cehj@naver.com (E.H.J.); clcn1004@naver.com (E.Y.); 2 Department of Anesthesiology and Pain Medicine, Hallym University Sacred Heart Hospital, Hallym University School of Medicine, Anyang 14068, Korea

**Keywords:** intraocular pressure, lateral decubitus position, ocular perfusion pressure, one-lung ventilation, positive end expiratory pressure, thoracoscopy

## Abstract

*Background and Objectives*: The effect of positive end-expiratory pressure (PEEP) on intraocular pressure (IOP) is debatable. There have been no studies investigating the effects of PEEP on IOP during one-lung ventilation (OLV). We aimed to investigate the effects of PEEP on IOP in patients undergoing OLV for video-assisted thoracoscopic surgery (VATs). *Materials and Methods*: Fifty-two patients undergoing VATs were divided into a zero-PEEP (ZEEP) and a 6 cmH_2_O of PEEP (PEEP) groups. IOP, ocular perfusion pressure (OPP), and respiratory and hemodynamic parameters were measured before induction (T1), immediately following endotracheal intubation (T2), 30 min (T3) and 60 min (T4) after a position change to the lateral decubitus position (LDP) and OLV, and 10 min following two-lung ventilation near the end of the surgery (T5). *Results*: There was no significant difference in IOP and OPP between the two groups. The IOP of the dependent eye was significantly higher than that of the non-dependent eye during LDP in both groups. Peak inspiratory pressure was significantly higher in the PEEP group than in the ZEEP group at T3–T5. Dynamic compliance was significantly higher in the PEEP group than in the ZEEP group at T2–T5. The ratio of arterial oxygen partial pressure to fractional inspired oxygen was significantly higher in the PEEP group than in the ZEEP group at T4. *Conclusions*: Applying 6 cmH_2_O of PEEP did not increase IOP but enhanced dynamic compliance and oxygenation during OLV. These results suggest that 6 cmH_2_O of PEEP can be safely applied during OLV in LDP.

## 1. Introduction

Intraocular pressure (IOP) is the pressure exerted by the contents of the eye on the lining of the eyeball. IOP can affect the integrity of the delicate structures that mediate vision [1]. An increase in IOP can compromise blood flow to the optic nerve and retina, resulting in a decrease in ocular perfusion pressure [1] and playing a role in the incidence of perioperative ophthalmic complications, such as ischemic optic neuropathy, retinal artery occlusion, and glaucoma [2]. Therefore, perioperative IOP monitoring may allow anesthesiologists to prevent the occurrence of ophthalmic complications.

Factors affecting perioperative IOP include the type and duration of operation, the surgical position, bleeding, blood pressure, airway pressure, colloid infusion, and anesthetics [1]. Among these factors, the surgical position can be one of the most important factors [1,3]. The surgical position can affect other factors related to IOP, such as blood pressure, airway pressure, and central venous pressure (CVP) [1]. Previous studies revealed that the IOP of the dependent eye can increase in the lateral decubitus position (LDP) in both awake and anesthetized patients [4,5,6]. A few cases of postoperative visual loss were reported in patients undergoing spine surgery in the LDP [7,8].

For lung surgery, patients are subjected to one-lung ventilation (OLV) of the dependent lung in the LDP. During OLV, peripheral oxygen saturation can be decreased due to ventilation-perfusion (V/Q) mismatch, hypoxic pulmonary vasoconstriction, and lung pathology [9]. Applying positive end-expiratory pressure (PEEP) to a ventilated lung can be helpful for managing hypoxia by increasing functional residual capacity and preventing alveolar collapse in patients undergoing OLV [9,10]. Previous studies have reported that PEEP may effect an IOP change; however, most of these studies were conducted in patients in the supine or head-up position with ventilation of two lungs (TLV) [11,12]. The LDP may cause IOP elevation of the dependent eye by a gravity effect [4], and PEEP may increase peak airway pressure and IOP. However, the effects of PEEP on IOP during OLV in the LDP have not yet been elucidated.

We hypothesized that it would be beneficial to apply PEEP if PEEP does not increase the IOP of the dependent eye in patients undergoing OLV in the LDP. Therefore, we aimed to compare the changes of IOP and respiratory parameters in patients undergoing OLV with or without PEEP in the LDP.

## 2. Materials and Methods

### 2.1. Study Design and Patient Selection

This study protocol was approved by the Institutional Review Board of Catholic University St. Vincent Hospital on 24 December 2020 (VC20OISI0251) and the randomized trial was registered at https://cris.nih.go.kr (accessed on 24 June 2021, KCT0006347). This manuscript adheres to the applicable CONSORT guidelines.

This single-center prospective randomized study was performed from January to October 2021 in a secondary university hospital in South Korea. A total of 52 patients, aged 30 to 75 years, with physical status I or II according to the American Society of Anesthesiologists (ASA) who were scheduled for an elective video-assisted thoracoscopic surgery ((VATs), lobectomy, segmentectomy, and wedge resection) were enrolled in this prospective, randomized, and controlled study, after obtaining written informed consent before the day of surgery. Patients with previous eye surgery or a preexisting eye disease, including glaucoma, uncontrolled cardiovascular disease, elevated intracranial pressure due to brain disease, poor pulmonary function (forced expiratory volume in 1 s/forced vital capacity % <60%), and baseline IOP ≥ 30 mmHg, were excluded. During surgery, patients with hemodynamic instability, massive bleeding (estimated blood loss > 500 mL), decreased peripheral oxygen saturation (SpO_2_) <93% during OLV, or an operation plan converted to a thoracotomy were withdrawn.

### 2.2. Randomization

On arrival to the operation theater, patients were divided into two groups, a zero-PEEP group (PEEP = 0, ZEEP) and a 6 cm H_2_O of PEEP group (PEEP) by randomization using the Research Randomizer (http://www.randomizer.org, accessed on 2 January 2021), with an allocation ratio of 1:1. The randomization was conducted by a resident anesthesiologist who was not involved in anesthetic management or data collection.

### 2.3. Anesthesia Protocol

All patients fasted for 8 h before the induction of anesthesia and none of the patients were premedicated. After arrival to the operation room, patients received basic monitoring, including electrocardiography, noninvasive blood pressure, SpO_2_, and bispectral index (BIS) and an IOP measuring device was prepared. After measuring baseline IOP, anesthesia was induced with 1.5–2.5 mg/kg of intravenous propofol and continuous infusion of remifentanil (0.1–0.2 μg/kg/min). Rocuronium (0.6 mg/kg) was administered to facilitate the insertion of a left-sided double-lumen tube into the trachea.

After endotracheal intubation, mechanical ventilation was started in a volume-controlled mode with an inspiratory/expiratory (I/E) ratio of 1:2 and a tidal volume of 8 mL/kg. PEEP was not applied in the ZEEP group whereas 6 cmH_2_O PEEP was applied in the PEEP group from the initiation of mechanical ventilation until the end of the surgery. The respiratory rate was adjusted to maintain an end-tidal carbon dioxide (CO_2_) pressure of 30–40 mmHg during anesthesia. Anesthesia was maintained with 5–8 vol% desflurane with 50% oxygen in the air and continuous infusion of remifentanil (0.02–0.15 μg/kg/min) to maintain a BIS of 40–60 and a systolic blood pressure within 20% of the initial value. During anesthesia, if the systolic blood pressure (SBP) was 160 mmHg or higher, 1 mg of nicardipine was administered intravenously. Tachycardia (heart rate (HR) > 110 bpm or HR increased by 30% from baseline) was treated with 10–20 mg of intravenous esmolol. Hypotension (SBP < 90 mmHg) was treated with 10 mg of intravenous ephedrine and bradycardia (HR < 50 bpm) with 0.2 mg of intravenous glycopyrrolate. Rocuronium was continuously infused to keep a train-of-four ratio between 1/4 and 2/4. Lactated Ringer’s solution was administered at a rate of 4–8 mL/kg/h during anesthesia.

After confirming the position of the double lumen tube with a bronchoscope, the patients were turned to the LDP. The head was maintained in a neutral position with a doughnut-shaped pillow and padding with towels. Immediately after the position change to the LDP, OLV was started with a tidal volume of 6 mL/kg. During OLV in the LDP, the dependent lung was ventilated with 80% oxygen. If the SpO_2_ was lower than 95%, the fraction of inspired oxygen (FiO_2_) was increased to 100%. When the surgical procedure was almost completed, patients were converted to TLV with a tidal volume of 8 mL/kg with 50% oxygen. After dressing their surgical wounds, patients were returned to a supine position. Following emergence from anesthesia, we asked patients about any ophthalmic complications they experienced, such as vision changes or eye discomfort.

### 2.4. Outcome Measures

The primary outcome was the change in IOP between the two groups and within each group. The secondary outcome we measured was change in ocular perfusion pressure (OPP) and respiratory parameters including peak inspiratory pressure (PIP), arterial partial pressure of oxygen (PaO_2_), PaO_2_/FiO_2_, and dynamic compliance (Cdyn).

The IOP was measured with a handheld tonometer (Tono-Pen AVIA, Reichert Technologies, Depew, NY, USA) after application of two drops of 0.5% Alcaine (proparacaine HCl 5mg, Alcon-Couvreur N.V., Puurs, Belgium). After applying topical anesthesia, the tonometer tip was placed perpendicular to the patient’s cornea and gently placed on the center of the cornea without causing indentation or additional pressure. IOP measurements were taken at five time points: before anesthetic induction in the supine position (T1, baseline), immediately following endotracheal intubation and mechanical ventilation (T2), 30 min after the position change to lateral decubitus, OLV (T3), 60 min after the position change to lateral decubitus, OLV (T4), and 10 min after TLV in the LDP, near the completion of the surgery (T5). The tonometer averaged readings from six successful measurements and displayed the mean value with a statistical confidence indicator. If the statistical confidence indicator was <95%, the value was discarded, and measurements were repeated. The IOP was measured by one anesthesiologist who had experience in measuring IOP from previous studies and did not participate in the data analysis.

At the time of each IOP measurement, we recorded mean arterial pressure (MAP), tidal volume, and PIP. The OPP was calculated as MAP minus IOP. Cdyn was calculated as tidal volume ÷ (PIP − PEEP). We performed arterial blood gas analysis at T2 and T4, and recorded PaO_2_, arterial partial pressure of CO_2_ (PaCO_2_), and PaO_2_/FiO_2_.

### 2.5. Statistical Analysis

The number of subjects required for each group was calculated with a power analysis based on a previous study of patients undergoing cholecystectomy [11], in which the mean IOP was approximately 17 mmHg in the zero PEEP group compared with 19 mmHg in the PEEP group, 5 min after the initiation of pneumoperitoneum. To detect a mean (±standard deviation) difference in the IOP of 2 ± 2.5 mmHg, the power estimation analysis suggested that 24 patients per group would be required to obtain a power of 80%, considering a type I error of 0.05. To compensate for unexpected losses, recruitment was increased by 10%.

Statistical analyses were performed using SPSS version 26.0 (SPSS Inc., Chicago, IL, USA) for Windows (Microsoft Corporation, Redmond, WA, USA). Demographic data were analyzed using the χ^2^ test and *t*-test. We performed a repeated-measures ANOVA to compare PaO_2_, PaCO_2_, PaO_2_/FiO_2_, PIP, Cdyn, MAP, IOP, and OPP between the two groups, with ‘group’ and ‘time point’ being the independent variables, after confirming a normal distribution using the Shapiro–Wilk test (*p* > 0.05). Differences between the two groups were then computed using a *t*-test, followed by a Bonferroni post hoc test (adjusted *p* value for significance *p* < 0.025 for PaO_2_, PaCO_2_, and PaO_2_/FiO_2_; *p* < 0.0125 for PIP and Cdyn; *p* < 0.01 for MAP, IOP and OPP). The IOP values of the dependent and non-dependent eyes were compared using a *t*-test at the same time points for each group. Changes from baseline to later time points in IOP (T1 versus T2–T5) and PIP (T2 versus T3–T5) were analyzed with a paired *t*-test for each group. The incidence of OPP < 50 mmHg was analyzed with Fisher’s exact test. A *p* value < 0.05 was considered statistically significant.

## 3. Results

**Figure 1:**
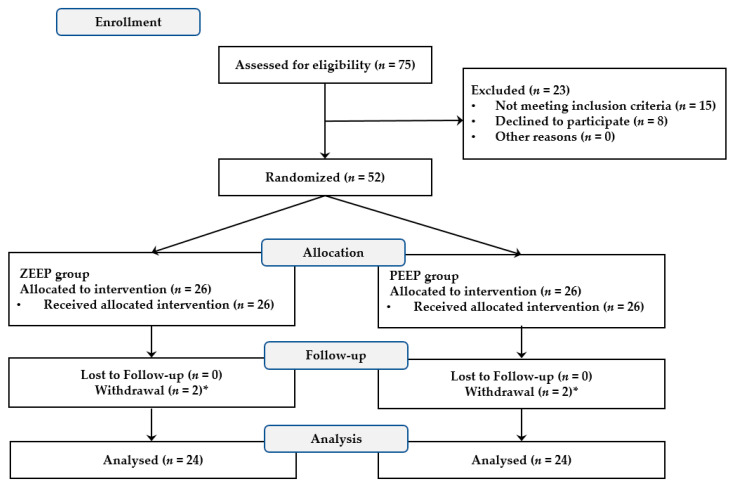
CONSORT flow diagram to illustrate the study design. * Two patients in the ZEEP group whose operation was altered to a thoracotomy and two patients in the PEEP group who had been transfused were withdrawn from the study.

A total of 52 patients were initially enrolled in this study, including 2 patients whose operations were altered to a thoracotomy in the ZEEP group. These two patients were excluded from the study, as were two patients in the PEEP group who had been transfused due to massive bleeding. As a result, 48 patients completed the study, with 24 each in the ZEEP and PEEP groups (Figure 1).

As shown in Table 1, the demographic and perioperative data were comparable between the two groups. During anesthesia, there were no significant differences in PaO_2_, PaCO_2_, and MAP between the two groups (Table 2). PaO_2_/FiO_2_ was comparable between the two groups at T2, but significantly higher in the PEEP group than in the ZEEP group at T4. PIP was significantly higher in the PEEP group than in the ZEEP group from T3 to T5. Compared with the initial PIP value (T2), PIP increased significantly during OLV (T3 and T4) in both groups (all *p* < 0.001). Cdyn was significantly higher in the PEEP group than in the ZEEP group at all time points (T2–T5).

There was no significant difference in IOP between the two groups during the study period (Table 3).

**Figure 2:**
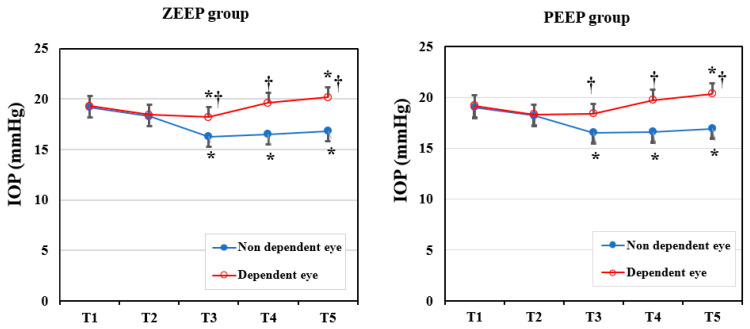
Changes in the intraocular pressure (IOP) in the ZEEP and PEEP groups (dependent eye vs. non-dependent eye). T1: before anesthetic induction in the supine position; T2: immediately after endotracheal intubation; T3: 30 min after position change to lateral decubitus and one-lung ventilation; T4: 60 min after position change to lateral decubitus and one-lung ventilation; T5: 10 min after two-lung ventilation in the LDP (near completion of surgery). * *p* < 0.05 compared with baseline (T1) value in the same eye. † *p* < 0.05 compared with the non-dependent eye.

To compare the IOP changes in dependent and non-dependent eyes, we performed an intragroup analysis (Figure 2). In each group, the IOP in the dependent eye was significantly higher than in the non-dependent eye after a position change to the LDP (ZEEP group: *p* = 0.008 in T3, *p* < 0.001 in T4 and T5; PEEP group: *p* = 0.013 in T3, *p* < 0.001 in T4 and T5). Changes in the IOP from the baseline value in each eye were similar in each group. The IOP in the non-dependent eye decreased significantly in T3–T5 compared with the baseline (all *p* < 0.001) in both groups. In the ZEEP group, the IOP of the dependent eye decreased significantly in T3 (*p* = 0.023), then increased significantly in T5 (*p* = 0.013), compared with the baseline value. In the PEEP group, the IOP of the dependent eye increased significantly in T5 (*p* = 0.04) relative to the baseline. There were no instances of severe ocular hypertension (IOP > 25 mmHg) during surgery.

As shown in Table 4, changes in OPP between the two groups were not significantly different. During the study period, OPP decreased by less than 50 mmHg in one patient (4.3%) and four patients (17.4%) in the ZEEP and PEEP groups, respectively; the incidence was not significantly different (*p* = 0.348).

No patient complained of any visual disturbance or discomfort after surgery in the recovery room.

## 4. Discussion

This study demonstrated that the application of 6 cmH_2_O of PEEP during OLV did not increase IOP nor decrease OPP significantly, but enhanced oxygenation and Cdyn. IOP in the dependent eye was higher than in the non-dependent eye during LDP in patients with or without PEEP. Compared with the initial value, IOP in the dependent eye increased 1 h after LDP, but IOP in the non-dependent eye decreased during LDP regardless of PEEP. This evidence suggests that PEEP cannot affect changes in IOP in the dependent eye during OLV.

During anesthesia, surgical factors, an underlying disease, and anesthetic management can be associated with a change in IOP [1]. Not only eye surgery, but general surgeries including laparoscopic, spinal, and cardiac procedures can induce an increase in IOP due to patient positioning and length of procedure, which can affect ocular perfusion [2]. In addition, patients who are elderly, have high IOP, glaucoma, or uncontrolled blood pressure may be vulnerable to perioperative ophthalmic complications [2]. Due to various lung diseases, including cancer, elderly patients with or without ophthalmic diseases are increasingly receiving VATs [13], and these patients need perioperative IOP management. Although patient conditions and surgical factors cannot be changed, the choice of anesthetic agents and ventilation mode are manageable factors that can prevent IOP elevation.

The LDP is known to be related to an increase in IOP in the dependent eye because of the gravity effect [4,6]. In this study, IOP in the non-dependent eye decreased 2–3 mmHg, but IOP in the dependent eye increased approximately 1 mmHg compared with the baseline value, 1 h after being placed in the LDP. In previous studies, the IOP of the dependent eye increased with time in the LDP, but the extent of change slightly differed depending on the anesthetic agent used or the patient’s blood pressure [4,6,14]. Yamada et al. included patients with TLV as well as OLV in their study and reported that the IOP in the dependent eye in the sevoflurane and propofol groups increased by 8 and 3–4 mmHg, respectively, from the baseline 1 h after placement in the LDP [6]. They also demonstrated that the IOP in the non-dependent eye 1 h after placement in the LDP did not change in the propofol group and increased about 4 mmHg from the baseline value in the sevoflurane group. Another study that involved patients undergoing OLV with sevoflurane reported that the IOP of the non-dependent eye was similar to the baseline value 1 h after placement in the LDP, whereas the IOP in the dependent eye increased by approximately 3–4 mmHg [4]. In hypotensive anesthesia in the LDP with TLV, the IOP of both eyes decreased but the IOP in the dependent eye decreased less than in the non-dependent eye [14]. Although the range of changes in the IOP of the dependent and non-dependent eyes was slightly different in previous studies, the difference in the IOP of both eyes was similar. The results of this study corresponded well with previous findings that reported that the difference of the IOP between dependent and non-dependent eyes was approximately 3–4 mmHg 1 h after placement in the LDP [4,6,14]. Based on our results, we can conclude that the LDP, rather than OLV itself, may influence IOP changes in the dependent eye.

During OLV, patients are prone to hypoxia due to V/Q mismatch and alveolar derecruitment, with reductions in the functional residual capacity of the dependent lung [15,16]. However, an increase in tidal volume or application of a high PEEP can cause an increase in airway pressure, leading to hyperinflation of the alveoli and stretching of the pulmonary parenchymal, which may result in lung injury [17,18,19]. Therefore, an appropriate combination of a PEEP with low tidal volume can play a protective role, reducing mechanical ventilation pressure and reducing postoperative complications [9,20]. To prevent acute lung injury, application of a low tidal volume (5–7 mL/kg) and a moderate level of PEEP (5–6 cmH_2_O) has been recommended in OLV [16]. As shown in the results of this study, 6 cmH_2_O of PEEP improves oxygenation and Cdyn. These results are consistent with earlier studies where Cdyn decreased more in OLV than in TLV, but PEEP alleviated this decrease [21,22].

The duration and pressure of PEEP may be related to increases in IOP [11,12]. The proposed mechanism for this is that an increased PIP can increase the CVP and disturb efflux blood from intraocular vessels, therefore elevating the IOP [12]. Teba et al. have proven that a high PEEP (≥15 mmHg) over a long duration is related to an increase in IOP in patients admitted to the intensive care unit [12]. However, only a few studies have examined the effects of PEEP on IOP in patients undergoing anesthesia and their results have been inconsistent. A previous study performed in patients undergoing cholecystectomy demonstrated that 10 cmH_2_O of PEEP did not increase IOP compared with baseline values, but when compared with the value at anesthesia induction, it significantly increased the IOP during pneumoperitoneum [11]. Another study showed that patients in the steep Trendelenburg position who received 5 cmH_2_O of PEEP did not exhibit higher IOP than patients without PEEP [23]. However, these previous studies were conducted in patients undergoing TLV in different positions and used procedures that were shorter in duration than that used in the present study. Studies investigating changes in IOP during OLV with PEEP remain limited. Our study showed that the IOP of patients with PEEP was comparable with the IOP of patients without PEEP during OLV.

In this study, when OLV was applied, the PIP increased significantly more than in TLV, regardless of PEEP, and the difference was about 2.5–3.5 cmH_2_O. Application of 6 cmH_2_O of PEEP was associated with an approximate 2 cmH_2_O increase in PIP. Because the tidal volume was reduced to 6 mL/kg during OLV, the difference in the PIP between the two groups was similar for TLV and OLV. These results were similar to previous studies that reported PIP increases of 2.5–5 cmH_2_O after OLV when a low tidal volume (6 mL/kg) and a PEEP of 5–8 cmH_2_O were applied [20,24]. Although we did not measure the CVP, we deduced that the reason why PEEP did not result in an increase in IOP was because an increase in PIP of ~2 cmH_2_O did not cause a significant increase in CVP.

In our study, the OPP values were comparable between the ZEEP and PEEP groups. Functional integrity of the retina is dependent on an adequate blood supply, and retinal and choroidal circulation feed the inner and outer layers of the retina, respectively [1]. Both choroidal and retinal circulation are autoregulated in response to a change in the OPP [1]. Although the critical OPP value at which retinal or optic nerve function is impaired has not yet been defined, a previous study has recommended a target physiological OPP range of 45–55 mmHg in patients with risk factors for ocular ischemia [1]. Previous studies have also suggested that the optic nerve flow becomes dysfunctional at an OPP of 30–35 mmHg [1,25]; therefore, maintenance of an adequate OPP during surgery may be important in patients at risk of postoperative ocular complications. In this study, OPP dropped continuously from baseline until the end of the surgery, but the lowest mean value was >65 mmHg in the dependent eye of both groups. This was likely because the increase in IOP was small and blood pressure was properly maintained in both groups. The incidence of OPP < 50 mmHg was slightly higher in the PEEP group than in the ZEEP group, but there was no significant difference. These findings also suggest that applying 6 cmH2O of PEEP has minimal or no effect on OPP.

There are a few limitations in this study. First, we did not record CVP because we did not insert a central venous catheter in all patients. A change in position to a prone or a steep head-down position, or a pneumoperitoneum are known to be main factors in IOP elevation, due to an increase in CVP [4,26]. If we had recorded CVP, we could have elucidated the changes of CVP by surgical pneumothorax and the relationship between IOP and CVP or PEEP and CVP. Second, we did not measure the urine output because we did not insert a foley catheter in patients undergoing a wedge resection. A positive fluid balance during anesthesia can be a reliable factor affecting IOP, but we could not calculate exact difference of input and output. Finally, PEEP was fixed at 6 cmH_2_O. Recent studies have recommended an individualized PEEP for maximum effect and minimal complications [21]. During OLV, we may need to apply higher PEEP than 6 cmH_2_O in patients with severe hypoxia who cannot be recovered with a high FiO_2_. If we conducted the study with a higher PEEP than 6 cmH_2_O or added more study groups, we may have had different results. Therefore, further study regarding individualized or higher PEEP and IOP change is needed for patients undergoing OLV.

## 5. Conclusions

In conclusion, the application of 6 cmH_2_O of PEEP did not result in increased IOP or decreased OPP in patients undergoing OLV in the LDP, but improved oxygenation and Cdyn. Therefore, 6 cmH_2_O of PEEP can be safely applied in patients undergoing OLV.

## Figures and Tables

**Table 1:** Demographic and perioperative data of study groups.

	Group ZEEP (*n* = 24)	Group PEEP (*n* = 24)	*p* Value
Age (year)	62.38 (10.25)	63.46 (7.67)	0.680
Sex (male/female)	12/12	15/9	0.383
Height (cm)	165.25 (7.33)	161.92(7.09)	0.116
Weight (kg)	66.17 (10.85)	61.46 (9.72)	0.12
ASA classification (1/2, *n*)	0/24	0/24	N/A
Smoker (*n*)	4	3	0.5
Hypertension (*n*)	10	11	0.771
Diabetes Mellitus (*n*)	7	9	0.540
Preoperative FEV_1_/FVC%	73.29 (4.37)	74.75 (4.30)	0.264
Type of surgery (*n* (%))			0.736
Lobectomy	13 (54.2)	14 (58.3)	
Segmentectomy	5 (20.8)	3 (12.5)	
Wedge resection	6 (25)	7 (29.2)	
Duration of anesthesia (min)	193.17 (83.64)	190.50 (48.99)	0.893
Duration of surgery (min)	138.29 (71.11)	128.83 (48.11)	0.592
Duration of OLV (min)	140.0 (75.37)	132.08 (41.73)	0.655
Fluid intake (mL)	1019.58 (531.68)	1054.17 (426.80)	0.805
Estimated blood loss (mL)	150.0 (165.68)	121.25 (64.63)	0.432
Number of patients receiving hemodynamic drugs during anesthesia (*n* (%))			
Nicardipine	8 (33.30)	10 (41.70)	0.551
Esmolol	11 (45.80)	14 (58.30)	0.386
Ephedrine	7 (29.20)	10 (41.70)	0.365
Glycopyrrolate	5 (20.80)	5 (20.80)	N/A

Number (%) or mean (Standard deviation). ASA: American Society of Anesthesiologists, FEV_1_: forced expiratory volume in one second, FVC%: forced vital capacity, OLV: One lung ventilation. N/A: not applicable.

**Table 2:** Respiratory and hemodynamic variables in the ZEEP and PEEP groups at each time point.

	Group ZEEP (*n* = 24)	Group PEEP (*n* = 24)	*p* Value
PaO_2_ (mmHg)			
T2	215.04 (47.96)	228.75 (45.77)	0.316
T4	130.88 (48.10)	160.17 (53.77)	0.053
PaCO_2_ (mmHg)			
T2	35.00 (2.81)	36.00 (3.80)	0.305
T4	37.92 (3.16)	36.58 (3.90)	0.200
PaO_2_/FiO_2_			
T2	430.16 (95.91)	457.50 (91.55)	0.317
T4	147.71 (54.91)	189.91 (69.06)	0.023
Peak inspiratory pressure (cmH_2_O)			
T2	16.46 (3.08)	18.29 (1.85)	0.016
T3	19.04 (2.39)	21.29 (1.57)	<0.001
T4	19.54 (2.25)	21.58 (1.79)	0.001
T5	15.21 (2.64)	18.67 (1.99)	<0.001
Dynamic compliance (mL/cmH_2_O)			
T2	30.46 (7.39)	38.29 (7.27)	0.001
T3	19.46 (3.69)	22.88 (3.49)	0.002
T4	18.88 (3.49)	22.79 (3.81)	0.001
T5	32.29 (6.67)	38.33 (7.41)	0.005
Mean arterial pressure (mmHg)			
T1	119.00 (11.85)	119.50 (13.47)	0.892
T2	118.92 (17.71)	119.88 (17.47)	0.851
T3	94.42 (12.79)	94.08 (11.28)	0.924
T4	88.50 (8.52)	87.54 (7.51)	0.681
T5	85.5 (8.49)	85.71 (7.14)	0.927

Mean (Standard deviation). PaO_2_: partial pressure of oxygen in arterial blood, PaCO_2_: partial pressure of carbon dioxide in arterial blood, FiO_2_: fraction of inspired oxygen. T1: before anesthetic induction in the supine position; T2: immediately after endotracheal intubation; T3: 30 min after position change to lateral decubitus and one-lung ventilation; T4: 60 min after position change to lateral decubitus and one-lung ventilation; T5: 10 min after two-lung ventilation in lateral decubitus.

**Table 3:** Comparisons of intraocular pressure (IOP) values between the study groups.

	T1	T2	T3	T4	T5
Non-dependent eye IOP (mmHg)					
ZEEP	19.17 (1.81)	18.29 (3.63)	16.29 (2.44)	16.50 (2.43)	16.83 (2.35)
PEEP	19.04 (2.63)	18.25 (2.66)	16.54 (2.57)	16.63 (2.31)	16.96 (2.29)
*p* value	0.849	0.964	0.731	0.856	0.853
Change from baseline					
ZEEP		−0.88 (2.99)	−2.88 (2.17)	−2.67 (2.14)	−2.33 (2.03)
PEEP		−0.79 (2.64)	−2.5 (2.90)	−2.42 (2.78)	−2.08 (2.67)
Dependent eye IOP (mmHg)					
ZEEP	19.29 (1.83)	18.42 (3.60)	18.21 (2.34)	19.63 (2.48)	20.17 (2.31)
PEEP	19.21 (2.83)	18.33 (2.62)	18.42 (2.48)	19.75 (2.47)	20.38 (2.37)
*p* value	0.904	0.927	0.766	0.862	0.905
Change from baseline					
ZEEP		−0.88 (2.65)	−1.08 (2.19)	0.33 (2.01)	0.87 (1.59)
PEEP		−0.88 (2.85)	−0.79 (3.23)	0.54 (2.78)	1.17 (2.63)

Mean (Standard deviation). ZEEP: ZEEP group, PEEP: PEEP group. T1: before anesthetic induction in the supine position; T2: immediately after endotracheal intubation; T3: 30 min after position change to lateral decubitus and one-lung ventilation; T4: 60 min after position change to lateral decubitus and one-lung ventilation; T5: 10 min after two-lung ventilation in lateral decubitus.

**Table 4:** Comparisons of ocular perfusion pressure (OPP) values.

	T1	T2	T3	T4	T5
Non-dependent eye OPP (mmHg)					
ZEEP	99.83 (11.93)	100.63 (16.18)	78.13 (13.13)	72.00 (8.42)	68.67 (8.48)
PEEP	100.46 (13.08)	101.62 (17.44)	77.54 (11.18)	70.92 (7.33)	68.75 (7.71)
*p* value	0.863	0.838	0.869	0.637	0.972
Dependent eye OPP (mmHg)					
ZEEP	99.71 (11.99)	100.50 (16.30)	76.21 (13.05)	68.88 (8.34)	65.25 (8.53)
PEEP	100.29 (13.03)	101.54 (17.57)	75.67 (11.23)	67.79 (7.37)	65.58 (7.77)
*p* value	0.873	0.832	0.878	0.636	0.888

Mean (standard deviation). T1: before anesthetic induction in the supine position; T2: immediately after endotracheal intubation; T3: 30 min after position change to lateral decubitus and one-lung ventilation; T4: 60 min after position change to lateral decubitus and one-lung ventilation; T5: 10 min after two-lung ventilation in lateral decubitus.

## Data Availability

The datasets used and/or analyzed during the current study are available from the corresponding author on reasonable request.
